# Supplementary material for: Enhancing the classification of isolated theropod teeth using machine learning: a comparative study
Source: PeerJ. 2025 Mar 26;13:e19116. doi: 10.7717/peerj.19116 (PMC11954464; doi:10.7717/peerj.19116)
Supplement: Supplemental Information 2 [file peerj-13-19116-s002.zip › data_preprocessing_visualization_of_correlation_taxa1.html]

Theropod Teeth- Genus level


Code 

- Show All Code
- Hide All Code

# Theropod Teeth- Genus level

#### Carolina Marques

#### 2025-01-07

# 1 Reading the data

```
data <- read_xlsx("Crown measurement dataset Kem Kem theropods.xlsx")
data<-data[data$`Taxa (Genus)`!="Abelisauridae",]
data[data == "?"] <- NA
data[data == "~"] <- NA
data[data == "/"] <- NA
# Remove "?" from all columns
data[] <- lapply(data, function(x) {
  gsub("\\? ","", x)
})

data[] <- lapply(data, function(x) {
  gsub("absent",0, x)
})

# Remove "?" from all columns
data[] <- lapply(data, function(x) {
  gsub("\\?","", x)
})


# Remove "?" from all columns
data[] <- lapply(data, function(x) {
  gsub("\\>","", x)
})

# Remove "?" from all columns
data[] <- lapply(data, function(x) {
  gsub("\\<","", x)
})
# Remove "?" from all columns
data[] <- lapply(data, function(x) {
  gsub(">","", x)
})

# Remove "?" from all columns
data[] <- lapply(data, function(x) {
  gsub("<","", x)
})
# Remove "?" from all columns
data[] <- lapply(data, function(x) {
  gsub("\\~","", x)
})

# Remove "?" from all columns
data[] <- lapply(data, function(x) {
  gsub("~","", x)
})

# Remove "?" from all columns
data[] <- lapply(data, function(x) {
  gsub(":","", x)
})
# Remove "?" from all columns
data[] <- lapply(data, function(x) {
  gsub(";","", x)
})
data$LIF<- ifelse(data$LIF=="6-7",6.5,
                  ifelse(data$LIF=="5-6",5.5,
                         ifelse(data$LIF=="4-5",4.5,
                                ifelse(data$LIF=="3-4","3.5",
                                                           ifelse(data$LIF=="11 or 12",11.5,
                                                                  ifelse(data$LIF=="10-13",12,data$LIF))))))
data$CH<-data$CH...22 
data<- data %>% select(-CH...60,-CH...22,-`(DDL/CH)*100`)
data1<-data
data<-data[,-c(1,2,5,4,6:14,16:19)]#until 19


data$`TransvUndu`<-ifelse(data$`Transv. Undu.`!=0 & !is.na(data$`Transv. Undu.`),1,data$`Transv. Undu.`)

data$`Interdentsulci`<-ifelse(data$`Interdent. sulci`!=0 & !is.na(data$`Interdent. sulci`),1,data$`Interdent. sulci`)

data$LAF<-ifelse(data$LAF=="6-7",6.5,data$LAF)

data$CTU1 <- sub(".*?(\\d+).*", "\\1", data$CTU)

data<- data %>% select(-CTU,-`Interdent. sulci`,-`Transv. Undu.`)

# Convert columns to numeric, then create log-transformed columns
data <- data %>%
  mutate(across(3:ncol(data), as.numeric)) %>%
  mutate(across(3:ncol(data), log, .names = "Log_{.col}"))

data$TaxonToothtype<-as.factor(data$TaxonToothtype)
#data$Epoch<-ifelse(data$Epoch=="'Middle Cretaceous'","Middle Cretaceous", data$Epoch)
#data$Epoch<-as.factor(data$Epoch)


data<-data.frame(data)
```

# 2 Checking the data

## 2.1 Summary of the table

```
summary(data)
```

```
##                    TaxonToothtype    Epoch                CBL        
##  Saurornitholestes lateral:133    Length:1349        Min.   : 0.380  
##  Tyrannosaurus lateral    :114    Class :character   1st Qu.: 4.205  
##  Acrocanthosaurus lateral : 48    Mode  :character   Median : 9.900  
##  Richardoestesia lateral  : 46                       Mean   :13.960  
##  Majungasaurus  lateral   : 41                       3rd Qu.:19.990  
##  Pectinodon lateral       : 40                       Max.   :54.500  
##  (Other)                  :927                       NA's   :3       
##       CBW               AL              CBR              CHR       
##  Min.   : 0.540   Min.   :  0.55   Min.   :0.2500   Min.   :0.400  
##  1st Qu.: 2.300   1st Qu.: 12.24   1st Qu.:0.4595   1st Qu.:1.632  
##  Median : 6.200   Median : 30.52   Median :0.5453   Median :1.905  
##  Mean   : 9.128   Mean   : 37.46   Mean   :0.5935   Mean   :1.933  
##  3rd Qu.:13.560   3rd Qu.: 55.81   3rd Qu.:0.6917   3rd Qu.:2.191  
##  Max.   :48.600   Max.   :152.84   Max.   :2.1841   Max.   :4.222  
##  NA's   :68       NA's   :338      NA's   :80       NA's   :13     
##       MCL              MCW              MCR              MDE         
##  Min.   : 0.320   Min.   : 0.940   Min.   :0.3841   Min.   :-13.880  
##  1st Qu.: 6.685   1st Qu.: 4.500   1st Qu.:0.5034   1st Qu.:  0.000  
##  Median :12.130   Median : 7.460   Median :0.5786   Median :  0.000  
##  Mean   :13.053   Mean   : 8.327   Mean   :0.6109   Mean   :  5.752  
##  3rd Qu.:18.015   3rd Qu.:11.050   3rd Qu.:0.6896   3rd Qu.:  8.592  
##  Max.   :37.100   Max.   :30.200   Max.   :1.2792   Max.   : 58.400  
##  NA's   :862      NA's   :892      NA's   :893      NA's   :1009     
##       MSL              MEC              LAF              LIF         
##  Min.   :  1.42   Min.   :  0.00   Min.   : 0.000   Min.   : 0.0000  
##  1st Qu.: 17.41   1st Qu.: 68.65   1st Qu.: 0.000   1st Qu.: 0.0000  
##  Median : 28.07   Median :100.00   Median : 0.000   Median : 0.0000  
##  Mean   : 32.88   Mean   : 83.59   Mean   : 0.407   Mean   : 0.4899  
##  3rd Qu.: 44.37   3rd Qu.:100.00   3rd Qu.: 0.000   3rd Qu.: 0.0000  
##  Max.   :123.63   Max.   :113.69   Max.   :15.000   Max.   :15.0000  
##  NA's   :1010     NA's   :1016     NA's   :736      NA's   :752      
##       DMT             DDT              DLAT            DLIT     
##  Min.   :0.100   Min.   : 0.100   Min.   :0.100   Min.   :0.10  
##  1st Qu.:1.400   1st Qu.: 1.200   1st Qu.:1.100   1st Qu.:1.10  
##  Median :2.140   Median : 3.200   Median :2.700   Median :2.26  
##  Mean   :2.900   Mean   : 3.191   Mean   :2.695   Mean   :2.48  
##  3rd Qu.:4.492   3rd Qu.: 4.350   3rd Qu.:4.060   3rd Qu.:3.39  
##  Max.   :8.500   Max.   :10.320   Max.   :8.140   Max.   :7.95  
##  NA's   :1303    NA's   :1304     NA's   :1302    NA's   :1304  
##        CA             CA2                MA              MC       
##  Min.   : 8.50   Min.   :-1.1200   Min.   : 4.66   Min.   : 4.70  
##  1st Qu.:68.27   1st Qu.:-0.0800   1st Qu.: 9.00   1st Qu.: 9.16  
##  Median :83.26   Median : 0.0100   Median :11.10   Median :12.00  
##  Mean   :74.85   Mean   : 0.0016   Mean   :13.75   Mean   :16.16  
##  3rd Qu.:86.34   3rd Qu.: 0.1000   3rd Qu.:14.00   3rd Qu.:19.00  
##  Max.   :88.11   Max.   : 0.3600   Max.   :60.00   Max.   :57.90  
##  NA's   :986     NA's   :1007      NA's   :873     NA's   :584    
##        MB              DA              DC              DB       
##  Min.   : 6.00   Min.   : 4.00   Min.   : 0.00   Min.   : 6.00  
##  1st Qu.:11.00   1st Qu.: 9.50   1st Qu.:10.03   1st Qu.:11.50  
##  Median :13.00   Median :12.00   Median :15.00   Median :14.60  
##  Mean   :14.25   Mean   :13.91   Mean   :17.87   Mean   :16.51  
##  3rd Qu.:16.00   3rd Qu.:15.00   3rd Qu.:21.25   3rd Qu.:18.56  
##  Max.   :45.00   Max.   :71.00   Max.   :70.00   Max.   :80.00  
##  NA's   :997     NA's   :776     NA's   :187     NA's   :829    
##       MAVG             DAVG           DAVG2             TDD        
##  Min.   : 0.000   Min.   : 1.56   Min.   :-0.920   Min.   :  0.20  
##  1st Qu.: 9.195   1st Qu.: 7.50   1st Qu.:-0.090   1st Qu.: 35.95  
##  Median :11.854   Median :11.50   Median : 0.011   Median : 57.19  
##  Mean   :14.457   Mean   :13.71   Mean   : 0.899   Mean   : 77.80  
##  3rd Qu.:16.144   3rd Qu.:16.25   3rd Qu.: 0.130   3rd Qu.:108.64  
##  Max.   :55.000   Max.   :80.00   Max.   :35.000   Max.   :368.62  
##  NA's   :633      NA's   :272     NA's   :1037     NA's   :281     
##       DSDI               CMA              CAA              CDA        
##  Min.   :  0.6654   Min.   : 16.11   Min.   : 3.151   Min.   : 17.79  
##  1st Qu.:  0.9322   1st Qu.: 58.10   1st Qu.:23.475   1st Qu.: 80.06  
##  Median :  1.0000   Median : 64.80   Median :27.060   Median : 87.06  
##  Mean   :  3.8502   Mean   : 64.54   Mean   :27.313   Mean   : 88.15  
##  3rd Qu.:  1.1167   3rd Qu.: 71.52   3rd Qu.:31.113   3rd Qu.: 94.38  
##  Max.   :269.8500   Max.   :148.96   Max.   :74.262   Max.   :160.74  
##  NA's   :730        NA's   :432      NA's   :427      NA's   :428     
##       MDL              DDL              ...61            CH        
##  Min.   :0.0864   Min.   :0.07143   Min.   : NA    Min.   :  0.57  
##  1st Qu.:0.2632   1st Qu.:0.23529   1st Qu.: NA    1st Qu.:  7.60  
##  Median :0.4167   Median :0.33333   Median : NA    Median : 18.08  
##  Mean   :0.4079   Mean   :0.36173   Mean   :NaN    Mean   : 28.11  
##  3rd Qu.:0.5459   3rd Qu.:0.49384   3rd Qu.: NA    3rd Qu.: 41.14  
##  Max.   :1.0638   Max.   :1.11111   Max.   : NA    Max.   :145.55  
##  NA's   :584      NA's   :187       NA's   :1349   NA's   :6       
##    TransvUndu     Interdentsulci        CTU1           Log_CBL       
##  Min.   :0.0000   Min.   :0.0000   Min.   : 0.000   Min.   :-0.9676  
##  1st Qu.:0.0000   1st Qu.:0.0000   1st Qu.: 0.000   1st Qu.: 1.4363  
##  Median :1.0000   Median :0.0000   Median : 2.000   Median : 2.2925  
##  Mean   :0.5215   Mean   :0.4162   Mean   : 1.513   Mean   : 2.1891  
##  3rd Qu.:1.0000   3rd Qu.:1.0000   3rd Qu.: 3.000   3rd Qu.: 2.9952  
##  Max.   :1.0000   Max.   :1.0000   Max.   :10.000   Max.   : 3.9982  
##  NA's   :954      NA's   :979      NA's   :953      NA's   :3        
##     Log_CBW            Log_AL           Log_CBR           Log_CHR       
##  Min.   :-0.6162   Min.   :-0.5978   Min.   :-1.3863   Min.   :-0.9163  
##  1st Qu.: 0.8329   1st Qu.: 2.5051   1st Qu.:-0.7777   1st Qu.: 0.4895  
##  Median : 1.8245   Median : 3.4184   Median :-0.6064   Median : 0.6444  
##  Mean   : 1.7245   Mean   : 3.1987   Mean   :-0.5725   Mean   : 0.6328  
##  3rd Qu.: 2.6071   3rd Qu.: 4.0220   3rd Qu.:-0.3685   3rd Qu.: 0.7842  
##  Max.   : 3.8836   Max.   : 5.0294   Max.   : 0.7812   Max.   : 1.4404  
##  NA's   :68        NA's   :338       NA's   :80        NA's   :13       
##     Log_MCL          Log_MCW           Log_MCR           Log_MDE     
##  Min.   :-1.139   Min.   :-0.0619   Min.   :-0.9570   Min.   : -Inf  
##  1st Qu.: 1.900   1st Qu.: 1.5041   1st Qu.:-0.6865   1st Qu.: -Inf  
##  Median : 2.496   Median : 2.0096   Median :-0.5471   Median : -Inf  
##  Mean   : 2.335   Mean   : 1.9125   Mean   :-0.5191   Mean   : -Inf  
##  3rd Qu.: 2.891   3rd Qu.: 2.4024   3rd Qu.:-0.3717   3rd Qu.:2.184  
##  Max.   : 3.614   Max.   : 3.4078   Max.   : 0.2462   Max.   :4.067  
##  NA's   :862      NA's   :892       NA's   :893       NA's   :1029   
##     Log_MSL          Log_MEC         Log_LAF         Log_LIF     
##  Min.   :0.3507   Min.   : -Inf   Min.   : -Inf   Min.   : -Inf  
##  1st Qu.:2.8568   1st Qu.:4.229   1st Qu.: -Inf   1st Qu.: -Inf  
##  Median :3.3347   Median :4.605   Median : -Inf   Median : -Inf  
##  Mean   :3.2561   Mean   : -Inf   Mean   : -Inf   Mean   : -Inf  
##  3rd Qu.:3.7926   3rd Qu.:4.605   3rd Qu.: -Inf   3rd Qu.: -Inf  
##  Max.   :4.8173   Max.   :4.734   Max.   :2.708   Max.   :2.708  
##  NA's   :1010     NA's   :1016    NA's   :736     NA's   :752    
##     Log_DMT           Log_DDT           Log_DLAT          Log_DLIT      
##  Min.   :-2.3026   Min.   :-2.3026   Min.   :-2.3026   Min.   :-2.3026  
##  1st Qu.: 0.3365   1st Qu.: 0.1823   1st Qu.: 0.0912   1st Qu.: 0.0953  
##  Median : 0.7604   Median : 1.1632   Median : 0.9933   Median : 0.8154  
##  Mean   : 0.7138   Mean   : 0.7984   Mean   : 0.5972   Mean   : 0.5271  
##  3rd Qu.: 1.5024   3rd Qu.: 1.4702   3rd Qu.: 1.4011   3rd Qu.: 1.2208  
##  Max.   : 2.1401   Max.   : 2.3341   Max.   : 2.0968   Max.   : 2.0732  
##  NA's   :1303      NA's   :1304      NA's   :1302      NA's   :1304     
##      Log_CA         Log_CA2           Log_MA          Log_MC     
##  Min.   :2.140   Min.   :  -Inf   Min.   :1.539   Min.   :1.548  
##  1st Qu.:4.223   1st Qu.:-3.219   1st Qu.:2.197   1st Qu.:2.215  
##  Median :4.422   Median :-2.408   Median :2.407   Median :2.485  
##  Mean   :4.272   Mean   :  -Inf   Mean   :2.495   Mean   :2.631  
##  3rd Qu.:4.458   3rd Qu.:-1.897   3rd Qu.:2.639   3rd Qu.:2.944  
##  Max.   :4.479   Max.   :-1.022   Max.   :4.094   Max.   :4.059  
##  NA's   :986     NA's   :1163     NA's   :873     NA's   :584    
##      Log_MB          Log_DA          Log_DC          Log_DB     
##  Min.   :1.792   Min.   :1.386   Min.   : -Inf   Min.   :1.792  
##  1st Qu.:2.398   1st Qu.:2.251   1st Qu.:2.305   1st Qu.:2.442  
##  Median :2.565   Median :2.485   Median :2.708   Median :2.681  
##  Mean   :2.608   Mean   :2.527   Mean   : -Inf   Mean   :2.721  
##  3rd Qu.:2.773   3rd Qu.:2.708   3rd Qu.:3.056   3rd Qu.:2.921  
##  Max.   :3.807   Max.   :4.263   Max.   :4.248   Max.   :4.382  
##  NA's   :997     NA's   :776     NA's   :187     NA's   :829    
##     Log_MAVG        Log_DAVG        Log_DAVG2         Log_TDD      
##  Min.   : -Inf   Min.   :0.4447   Min.   :  -Inf   Min.   :-1.609  
##  1st Qu.:2.219   1st Qu.:2.0149   1st Qu.:-2.996   1st Qu.: 3.582  
##  Median :2.473   Median :2.4423   Median :-2.263   Median : 4.046  
##  Mean   : -Inf   Mean   :2.3736   Mean   :  -Inf   Mean   : 4.038  
##  3rd Qu.:2.781   3rd Qu.:2.7881   3rd Qu.:-1.561   3rd Qu.: 4.688  
##  Max.   :4.007   Max.   :4.3820   Max.   : 3.555   Max.   : 5.910  
##  NA's   :633     NA's   :272      NA's   :1174     NA's   :281     
##     Log_DSDI          Log_CMA         Log_CAA         Log_CDA     
##  Min.   :-0.4074   Min.   :2.779   Min.   :1.148   Min.   :2.879  
##  1st Qu.:-0.0702   1st Qu.:4.062   1st Qu.:3.156   1st Qu.:4.383  
##  Median : 0.0000   Median :4.171   Median :3.298   Median :4.467  
##  Mean   : 0.1043   Mean   :4.149   Mean   :3.274   Mean   :4.467  
##  3rd Qu.: 0.1104   3rd Qu.:4.270   3rd Qu.:3.438   3rd Qu.:4.547  
##  Max.   : 5.5979   Max.   :5.004   Max.   :4.308   Max.   :5.080  
##  NA's   :730       NA's   :432     NA's   :427     NA's   :428    
##     Log_MDL           Log_DDL          Log_...61        Log_CH       
##  Min.   :-2.4493   Min.   :-2.6391   Min.   : NA    Min.   :-0.5621  
##  1st Qu.:-1.3350   1st Qu.:-1.4469   1st Qu.: NA    1st Qu.: 2.0281  
##  Median :-0.8755   Median :-1.0986   Median : NA    Median : 2.8948  
##  Mean   :-1.0214   Mean   :-1.1344   Mean   :NaN    Mean   : 2.8269  
##  3rd Qu.:-0.6054   3rd Qu.:-0.7056   3rd Qu.: NA    3rd Qu.: 3.7170  
##  Max.   : 0.0619   Max.   : 0.1054   Max.   : NA    Max.   : 4.9805  
##  NA's   :584       NA's   :187       NA's   :1349   NA's   :6        
##  Log_TransvUndu Log_Interdentsulci    Log_CTU1     
##  Min.   :-Inf   Min.   :-Inf       Min.   :  -Inf  
##  1st Qu.:-Inf   1st Qu.:-Inf       1st Qu.:  -Inf  
##  Median :   0   Median :-Inf       Median :0.6931  
##  Mean   :-Inf   Mean   :-Inf       Mean   :  -Inf  
##  3rd Qu.:   0   3rd Qu.:   0       3rd Qu.:1.0986  
##  Max.   :   0   Max.   :   0       Max.   :2.3026  
##  NA's   :954    NA's   :979        NA's   :953
```

## 2.2 Check first rows

```
head(data)
```

## 2.3 Tables

### 2.3.1 Genus / Taxa Table

```
taxa<-table(data$TaxonToothtype)
data_taxa<-data.frame(taxa)
data_taxa<-data_taxa[order(data_taxa$Freq, decreasing = T), ]
data_taxa$ID<-1:nrow(data_taxa)
data_taxa
```

# 3 Data Processing

## 3.1 Removing columns that have more more missing value

```
# Count the number of missing values in each column
missing_counts <- colSums(is.na(data))

# Remove columns with more than 15% missing values
data1_cleaned <- data[, missing_counts <= nrow(data)*0.15]
```

## 3.2 Removing rows with NA values

```
# Remove rows with any NA values
data1_cleaned <- na.omit(data1_cleaned)
data1_cleaned
```

## 3.3 Subtracting the observations for the Taxa that have least observations

```
lennn<-(ncol(data1_cleaned)-2)/2
taxa1<-table(data1_cleaned$TaxonToothtype)
data1_cleanedd<-data.frame(taxa1)
data1_cleanedd<-data1_cleanedd[order(data1_cleanedd$Freq, decreasing = T), ]
data1_cleanedd$ID<-1:nrow(data1_cleanedd)
data1_cleanedd$TaxonToothtype<-data1_cleanedd$Var1

data1_cleaned1<-data1_cleanedd[data1_cleanedd$Freq>lennn,]

data1_cleaned<-data1_cleaned[data1_cleaned$TaxonToothtype%in%unique(data1_cleaned1$TaxonToothtype),]

summary(data1_cleaned)
```

```
##                    TaxonToothtype    Epoch                CBL       
##  Saurornitholestes lateral:133    Length:931         Min.   : 1.40  
##  Tyrannosaurus lateral    :106    Class :character   1st Qu.: 4.74  
##  Richardoestesia lateral  : 45    Mode  :character   Median :11.25  
##  Acrocanthosaurus lateral : 42                       Mean   :15.22  
##  Majungasaurus  lateral   : 40                       3rd Qu.:22.56  
##  Pectinodon lateral       : 40                       Max.   :54.50  
##  (Other)                  :525                                      
##       CBW              CBR              CHR              DC       
##  Min.   : 0.600   Min.   :0.2500   Min.   :0.400   Min.   : 4.50  
##  1st Qu.: 2.200   1st Qu.:0.4473   1st Qu.:1.632   1st Qu.:10.00  
##  Median : 5.800   Median :0.5206   Median :1.903   Median :15.00  
##  Mean   : 9.451   Mean   :0.5682   Mean   :1.911   Mean   :17.40  
##  3rd Qu.:14.495   3rd Qu.:0.6531   3rd Qu.:2.172   3rd Qu.:20.25  
##  Max.   :48.600   Max.   :2.1840   Max.   :3.509   Max.   :60.00  
##                                                                   
##       DDL                CH             Log_CBL          Log_CBW       
##  Min.   :0.08333   Min.   :  2.200   Min.   :0.3365   Min.   :-0.5108  
##  1st Qu.:0.24692   1st Qu.:  8.425   1st Qu.:1.5560   1st Qu.: 0.7885  
##  Median :0.33333   Median : 18.810   Median :2.4204   Median : 1.7579  
##  Mean   :0.36742   Mean   : 30.574   Mean   :2.3168   Mean   : 1.7015  
##  3rd Qu.:0.50000   3rd Qu.: 45.535   3rd Qu.:3.1162   3rd Qu.: 2.6738  
##  Max.   :1.11111   Max.   :145.550   Max.   :3.9982   Max.   : 3.8836  
##                                                                        
##     Log_CBR           Log_CHR            Log_DC         Log_DDL       
##  Min.   :-1.3863   Min.   :-0.9163   Min.   :1.504   Min.   :-2.4849  
##  1st Qu.:-0.8045   1st Qu.: 0.4897   1st Qu.:2.303   1st Qu.:-1.3987  
##  Median :-0.6527   Median : 0.6436   Median :2.708   Median :-1.0986  
##  Mean   :-0.6141   Mean   : 0.6248   Mean   :2.725   Mean   :-1.1153  
##  3rd Qu.:-0.4261   3rd Qu.: 0.7755   3rd Qu.:3.008   3rd Qu.:-0.6931  
##  Max.   : 0.7812   Max.   : 1.2554   Max.   :4.094   Max.   : 0.1054  
##                                                                       
##      Log_CH      
##  Min.   :0.7885  
##  1st Qu.:2.1312  
##  Median :2.9344  
##  Mean   :2.9493  
##  3rd Qu.:3.8185  
##  Max.   :4.9805  
##
```

## 3.4 Spliting the Log variables and the original

```
# Select variables that contain "log" and the first column
selected_cols <- c(1,2, grep("Log", names(data1_cleaned)))

# Subset the data frame
data_log <- data1_cleaned[, selected_cols]

data_log
```

```
names(data_log)[-1]  <- gsub(" ", "_", names(data_log)[-1] )


# Identify columns that contain "log"
log_cols <- grep("Log", names(data1_cleaned))

# Include the first column
cols_to_keep <- setdiff(1:ncol(data1_cleaned), log_cols)

# Ensure the first column is included
cols_to_keep <- union(1, cols_to_keep)

# Subset the data frame
data_original <- data1_cleaned[, cols_to_keep]

data_original
```

# 4 Visualization

## 4.1 Original data

```
correlation_matrix <- cor(data_original[, -c(1, 2)])
# Plot correlation matrix
corrplot(correlation_matrix, method = "color", type = "lower", 
         addCoef.col = "black", 
         tl.col = "black", 
         tl.srt = 45, 
         diag = FALSE, 
         order = "hclust", 
         col = colorRampPalette(c("blue", "white", "red"))(200))
```

## 4.2 Log data

```
correlation_matrix <- cor(data_log[, -c(1, 2)])
# Plot correlation matrix
corrplot(correlation_matrix, method = "color", type = "lower", 
         addCoef.col = "black", 
         tl.col = "black", 
         tl.srt = 45, 
         diag = FALSE, 
         order = "hclust", 
         col = colorRampPalette(c("blue", "white", "red"))(200))
```

```
colnames(data_original)
```

```
## [1] "TaxonToothtype" "Epoch"          "CBL"            "CBW"           
## [5] "CBR"            "CHR"            "DC"             "DDL"           
## [9] "CH"
```

```
# Filter out taxa with less than 10 observations
data_log <- data_log %>%
  group_by(TaxonToothtype) %>%                   # Group by 'Taxa' column
  filter(n() >= 10) %>%                # Keep only groups with 10 or more observations
  ungroup()                            # Ungroup after filtering
# Filter out taxa with less than 10 observations
data_original <- data_original %>%
  group_by(TaxonToothtype) %>%                   # Group by 'Taxa' column
  filter(n() >= 10) %>%                # Keep only groups with 10 or more observations
  ungroup() 
# Get the count of each unique value in the column
category_counts <- table(data_log$TaxonToothtype)

# Filter unique values that have more than 0 observations
unique_values <- names(category_counts[category_counts > 0])

# Print the result
#print(unique_values)
```

# 5 Merging data clade with tooth to add more classes

```
data1$TaxonToothtype<-ifelse(!(data1$TaxonToothtype%in%unique_values),data1$`Taxa (Genus)`,data1$TaxonToothtype)

data1<-data1[,-c(1,2,5,4,6:14,16:19)]#until 19
#clade: 4, taxa:2, teethtaxa: 3, cladetteth: 5, epoch:15
#data<-inner_join(dd,data)
#data<-data[!duplicated(data),]

data1$`TransvUndu`<-ifelse(data1$`Transv. Undu.`!=0 & !is.na(data1$`Transv. Undu.`),1,data1$`Transv. Undu.`)

data1$`Interdentsulci`<-ifelse(data1$`Interdent. sulci`!=0 & !is.na(data1$`Interdent. sulci`),1,data1$`Interdent. sulci`)

data1$LAF<-ifelse(data1$LAF=="6-7",6.5,data1$LAF)

data1$CTU1 <- sub(".*?(\\d+).*", "\\1", data1$CTU)

data1<- data1 %>% select(-CTU,-`Interdent. sulci`,-`Transv. Undu.`)

# Convert columns to numeric, then create log-transformed columns
data1 <- data1 %>%
  mutate(across(3:ncol(data1), as.numeric)) %>%
  mutate(across(3:ncol(data1), log, .names = "Log_{.col}"))
```

```
## Warning: There were 17 warnings in `mutate()`.
## The first warning was:
## ℹ In argument: `across(3:ncol(data1), as.numeric)`.
## Caused by warning:
## ! NAs introduced by coercion
## ℹ Run `dplyr::last_dplyr_warnings()` to see the 16 remaining warnings.
```

```
## Warning: There were 3 warnings in `mutate()`.
## The first warning was:
## ℹ In argument: `across(3:ncol(data1), log, .names = "Log_{.col}")`.
## Caused by warning:
## ! NaNs produced
## ℹ Run `dplyr::last_dplyr_warnings()` to see the 2 remaining warnings.
```

```
data1$TaxonToothtype<-as.factor(data1$TaxonToothtype)
#data1$Epoch<-ifelse(data1$Epoch=="'Middle Cretaceous'","Middle Cretaceous", data1$Epoch)
#data1$Epoch<-as.factor(data1$Epoch)


#data$Taxa<-as.factor(paste0(data$`Taxa (Genus)`,data$Maturity,sep=" "))
data1<-data.frame(data1)


# Count the number of missing values in each column
missing_counts <- colSums(is.na(data1))

# Remove columns with more than 15% missing values
data1_cleaned <- data1[, missing_counts <= nrow(data1)*0.15]

# Remove rows with any NA values
data1_cleaned <- na.omit(data1_cleaned)
data1_cleaned
```

```
lennn<-(ncol(data1_cleaned)-2)/2
taxa1<-table(data1_cleaned$TaxonToothtype)
data1_cleanedd<-data.frame(taxa1)
data1_cleanedd<-data1_cleanedd[order(data1_cleanedd$Freq, decreasing = T), ]
data1_cleanedd$ID<-1:nrow(data1_cleanedd)
data1_cleanedd$TaxonToothtype<-data1_cleanedd$Var1

data1_cleaned1<-data1_cleanedd[data1_cleanedd$Freq>lennn,]

data1_cleaned<-data1_cleaned[data1_cleaned$TaxonToothtype%in%unique(data1_cleaned1$TaxonToothtype),]

summary(data1_cleaned)
```

```
##                    TaxonToothtype    Epoch                CBL        
##  Saurornitholestes lateral:133    Length:968         Min.   : 1.400  
##  Tyrannosaurus lateral    :106    Class :character   1st Qu.: 4.815  
##  Richardoestesia lateral  : 45    Mode  :character   Median :11.310  
##  Acrocanthosaurus lateral : 42                       Mean   :15.179  
##  Majungasaurus  lateral   : 40                       3rd Qu.:22.348  
##  Pectinodon lateral       : 40                       Max.   :54.500  
##  (Other)                  :562                                       
##       CBW              CBR              CHR              DC       
##  Min.   : 0.600   Min.   :0.2500   Min.   :0.400   Min.   : 4.50  
##  1st Qu.: 2.200   1st Qu.:0.4489   1st Qu.:1.639   1st Qu.:10.00  
##  Median : 6.000   Median :0.5243   Median :1.913   Median :15.00  
##  Mean   : 9.408   Mean   :0.5710   Mean   :1.920   Mean   :17.33  
##  3rd Qu.:14.402   3rd Qu.:0.6590   3rd Qu.:2.181   3rd Qu.:20.00  
##  Max.   :48.600   Max.   :2.1840   Max.   :3.509   Max.   :60.00  
##                                                                   
##       DDL                CH            Log_CBL          Log_CBW       
##  Min.   :0.08333   Min.   :  2.20   Min.   :0.3365   Min.   :-0.5108  
##  1st Qu.:0.25000   1st Qu.:  8.50   1st Qu.:1.5717   1st Qu.: 0.7885  
##  Median :0.33333   Median : 19.84   Median :2.4257   Median : 1.7918  
##  Mean   :0.36710   Mean   : 30.54   Mean   :2.3218   Mean   : 1.7114  
##  3rd Qu.:0.50000   3rd Qu.: 45.19   3rd Qu.:3.1067   3rd Qu.: 2.6674  
##  Max.   :1.11111   Max.   :145.55   Max.   :3.9982   Max.   : 3.8836  
##                                                                       
##     Log_CBR           Log_CHR            Log_DC         Log_DDL       
##  Min.   :-1.3863   Min.   :-0.9163   Min.   :1.504   Min.   :-2.4849  
##  1st Qu.:-0.8010   1st Qu.: 0.4943   1st Qu.:2.303   1st Qu.:-1.3863  
##  Median :-0.6457   Median : 0.6487   Median :2.708   Median :-1.0986  
##  Mean   :-0.6093   Mean   : 0.6297   Mean   :2.723   Mean   :-1.1136  
##  3rd Qu.:-0.4171   3rd Qu.: 0.7799   3rd Qu.:2.996   3rd Qu.:-0.6931  
##  Max.   : 0.7812   Max.   : 1.2554   Max.   :4.094   Max.   : 0.1054  
##                                                                       
##      Log_CH      
##  Min.   :0.7885  
##  1st Qu.:2.1401  
##  Median :2.9880  
##  Mean   :2.9590  
##  3rd Qu.:3.8108  
##  Max.   :4.9805  
##
```

```
# Select variables that contain "log" and the first column
selected_cols <- c(1,2, grep("Log", names(data1_cleaned)))

# Subset the data frame
data_log <- data1_cleaned[, selected_cols]

data_log
```

```
names(data_log)[-1]  <- gsub(" ", "_", names(data_log)[-1] )


# Identify columns that contain "log"
log_cols <- grep("Log", names(data1_cleaned))

# Include the first column
cols_to_keep <- setdiff(1:ncol(data1_cleaned), log_cols)

# Ensure the first column is included
cols_to_keep <- union(1, cols_to_keep)

# Subset the data frame
data_original <- data1_cleaned[, cols_to_keep]

data_original
```

```
colnames(data_original)
```

```
## [1] "TaxonToothtype" "Epoch"          "CBL"            "CBW"           
## [5] "CBR"            "CHR"            "DC"             "DDL"           
## [9] "CH"
```

```
# Filter out taxa with less than 10 observations
data_log <- data_log %>%
  group_by(TaxonToothtype) %>%                   # Group by 'Taxa' column
  filter(n() >= 10) %>%                # Keep only groups with 10 or more observations
  ungroup()                            # Ungroup after filtering
# Filter out taxa with less than 10 observations
data_original <- data_original %>%
  group_by(TaxonToothtype) %>%                   # Group by 'Taxa' column
  filter(n() >= 10) %>%                # Keep only groups with 10 or more observations
  ungroup() 
# Get the count of each unique value in the column
category_counts <- table(data_log$TaxonToothtype)

# Filter unique values that have more than 0 observations
unique_values1 <- names(category_counts[category_counts > 0])
```

# 6 Saving the clean dataset

```
write.csv(data_log,"teeth_data_log_taxa_epoch1.csv", row.names = FALSE)
write.csv(data_original,"teeth_data_taxa_epoch1.csv", row.names = FALSE)
```
