# Supplementary material for: Enhancing the classification of isolated theropod teeth using machine learning: a comparative study
Source: PeerJ. 2025 Mar 26;13:e19116. doi: 10.7717/peerj.19116 (PMC11954464; doi:10.7717/peerj.19116)
Supplement: Supplemental Information 2 [file peerj-13-19116-s002.zip › data_preprocessing_visualization_of_correlation_clade1.html]

Theropod Teeth- Higher taxonomic level


Code 

- Show All Code
- Hide All Code

# Theropod Teeth- Higher taxonomic level

#### Carolina Marques

#### 2025-01-07

# 1 Reading the data

```
data <- read_xlsx("Crown measurement dataset Kem Kem theropods.xlsx")

data[data == "?"] <- NA
data[data == "~"] <- NA
data[data == "/"] <- NA
# Remove "?" from all columns
data[] <- lapply(data, function(x) {
  gsub("\\? ","", x)
})

data[] <- lapply(data, function(x) {
  gsub("absent",0, x)
})

# Remove "?" from all columns
data[] <- lapply(data, function(x) {
  gsub("\\?","", x)
})


# Remove "?" from all columns
data[] <- lapply(data, function(x) {
  gsub("\\>","", x)
})

# Remove "?" from all columns
data[] <- lapply(data, function(x) {
  gsub("\\<","", x)
})
# Remove "?" from all columns
data[] <- lapply(data, function(x) {
  gsub(">","", x)
})

# Remove "?" from all columns
data[] <- lapply(data, function(x) {
  gsub("<","", x)
})
# Remove "?" from all columns
data[] <- lapply(data, function(x) {
  gsub("\\~","", x)
})

# Remove "?" from all columns
data[] <- lapply(data, function(x) {
  gsub("~","", x)
})

# Remove "?" from all columns
data[] <- lapply(data, function(x) {
  gsub(":","", x)
})
# Remove "?" from all columns
data[] <- lapply(data, function(x) {
  gsub(";","", x)
})
data$LIF<- ifelse(data$LIF=="6-7",6.5,
                  ifelse(data$LIF=="5-6",5.5,
                         ifelse(data$LIF=="4-5",4.5,
                                ifelse(data$LIF=="3-4","3.5",
                                                           ifelse(data$LIF=="11 or 12",11.5,
                                                                  ifelse(data$LIF=="10-13",12,data$LIF))))))
data$CH<-data$CH...22 
data<- data %>% select(-CH...60,-CH...22,-`(DDL/CH)*100`)
data1<-data
data<-data[,-c(1,2,3,4,6:14,16:19)]#until 19

data$`TransvUndu`<-ifelse(data$`Transv. Undu.`!=0 & !is.na(data$`Transv. Undu.`),1,data$`Transv. Undu.`)

data$`Interdentsulci`<-ifelse(data$`Interdent. sulci`!=0 & !is.na(data$`Interdent. sulci`),1,data$`Interdent. sulci`)

data$LAF<-ifelse(data$LAF=="6-7",6.5,data$LAF)

data$CTU1 <- sub(".*?(\\d+).*", "\\1", data$CTU)

data<- data %>% select(-CTU,-`Interdent. sulci`,-`Transv. Undu.`)

# Convert columns to numeric, then create log-transformed columns
data <- data %>%
  mutate(across(3:ncol(data), as.numeric)) %>%
  mutate(across(3:ncol(data), log, .names = "Log_{.col}"))

data$CladeToothtype<-as.factor(data$CladeToothtype)
#data$Epoch<-ifelse(data$Epoch=="'Middle Cretaceous'","Middle Cretaceous", data$Epoch)
#data$Epoch<-as.factor(data$Epoch)


data<-data.frame(data)
```

# 2 Checking the data

## 2.1 Summary of the table

```
summary(data)
```

```
##                                  CladeToothtype    Epoch          
##  Dromaeosauridae Lateral                :317    Length:1371       
##  Tyrannosauridae Lateral                :185    Class :character  
##  Carcharodontosauridae Lateral          : 85    Mode  :character  
##  Troodontidae Lateral                   : 81                      
##  Abelisauridae Lateral                  : 73                      
##  Non-spinosaurid Megalosauroidea Lateral: 72                      
##  (Other)                                :558                      
##       CBL              CBW               AL              CBR        
##  Min.   : 0.380   Min.   : 0.540   Min.   :  0.55   Min.   :0.2500  
##  1st Qu.: 4.282   1st Qu.: 2.300   1st Qu.: 12.40   1st Qu.:0.4598  
##  Median : 9.950   Median : 6.100   Median : 29.82   Median :0.5420  
##  Mean   :13.916   Mean   : 9.074   Mean   : 37.19   Mean   :0.5920  
##  3rd Qu.:19.782   3rd Qu.:13.430   3rd Qu.: 55.15   3rd Qu.:0.6895  
##  Max.   :54.500   Max.   :48.600   Max.   :152.84   Max.   :2.1841  
##  NA's   :3        NA's   :69       NA's   :338      NA's   :81      
##       CHR             MCL             MCW              MCR        
##  Min.   :0.400   Min.   : 0.32   Min.   : 0.940   Min.   :0.3841  
##  1st Qu.:1.634   1st Qu.: 6.69   1st Qu.: 4.480   1st Qu.:0.5000  
##  Median :1.908   Median :12.13   Median : 7.390   Median :0.5745  
##  Mean   :1.935   Mean   :13.02   Mean   : 8.274   Mean   :0.6087  
##  3rd Qu.:2.191   3rd Qu.:17.98   3rd Qu.:10.960   3rd Qu.:0.6818  
##  Max.   :4.222   Max.   :37.10   Max.   :30.200   Max.   :1.2792  
##  NA's   :14      NA's   :876     NA's   :906      NA's   :907     
##       MDE               MSL              MEC              LAF         
##  Min.   :-13.880   Min.   :  1.42   Min.   :  0.00   Min.   : 0.0000  
##  1st Qu.:  0.000   1st Qu.: 17.55   1st Qu.: 68.74   1st Qu.: 0.0000  
##  Median :  0.000   Median : 28.02   Median :100.00   Median : 0.0000  
##  Mean   :  5.636   Mean   : 32.77   Mean   : 83.93   Mean   : 0.3948  
##  3rd Qu.:  8.360   3rd Qu.: 44.01   3rd Qu.:100.00   3rd Qu.: 0.0000  
##  Max.   : 58.400   Max.   :123.63   Max.   :113.69   Max.   :15.0000  
##  NA's   :1024      NA's   :1025     NA's   :1031     NA's   :739      
##       LIF               DMT             DDT              DLAT      
##  Min.   : 0.0000   Min.   :0.100   Min.   : 0.100   Min.   :0.100  
##  1st Qu.: 0.0000   1st Qu.:1.400   1st Qu.: 1.250   1st Qu.:1.000  
##  Median : 0.0000   Median :2.200   Median : 3.000   Median :2.400  
##  Mean   : 0.4748   Mean   :2.888   Mean   : 3.152   Mean   :2.625  
##  3rd Qu.: 0.0000   3rd Qu.:4.485   3rd Qu.: 4.255   3rd Qu.:4.000  
##  Max.   :15.0000   Max.   :8.500   Max.   :10.320   Max.   :8.140  
##  NA's   :755       NA's   :1324    NA's   :1324     NA's   :1322   
##       DLIT             CA             CA2               MA       
##  Min.   :0.100   Min.   : 8.50   Min.   :-1.120   Min.   : 4.66  
##  1st Qu.:1.075   1st Qu.:68.27   1st Qu.:-0.080   1st Qu.: 9.00  
##  Median :2.200   Median :83.22   Median : 0.010   Median :11.25  
##  Mean   :2.432   Mean   :74.87   Mean   : 0.002   Mean   :13.76  
##  3rd Qu.:3.345   3rd Qu.:86.28   3rd Qu.: 0.100   3rd Qu.:14.00  
##  Max.   :7.950   Max.   :88.11   Max.   : 0.360   Max.   :60.00  
##  NA's   :1324    NA's   :1004    NA's   :1026     NA's   :888    
##        MC              MB              DA              DC              DB      
##  Min.   : 4.70   Min.   : 6.00   Min.   : 4.00   Min.   : 0.00   Min.   : 6.0  
##  1st Qu.: 9.25   1st Qu.:11.00   1st Qu.: 9.50   1st Qu.:10.24   1st Qu.:11.5  
##  Median :12.00   Median :13.00   Median :12.00   Median :15.00   Median :14.8  
##  Mean   :16.09   Mean   :14.25   Mean   :13.89   Mean   :17.79   Mean   :16.5  
##  3rd Qu.:19.00   3rd Qu.:16.00   3rd Qu.:15.00   3rd Qu.:21.06   3rd Qu.:18.5  
##  Max.   :57.90   Max.   :45.00   Max.   :71.00   Max.   :70.00   Max.   :80.0  
##  NA's   :590     NA's   :1015    NA's   :790     NA's   :191     NA's   :845   
##       MAVG            DAVG           DAVG2              TDD        
##  Min.   : 0.00   Min.   : 1.56   Min.   :-0.9200   Min.   :  0.20  
##  1st Qu.: 9.20   1st Qu.: 7.65   1st Qu.:-0.0940   1st Qu.: 36.00  
##  Median :12.00   Median :11.50   Median : 0.0100   Median : 56.80  
##  Mean   :14.44   Mean   :13.69   Mean   : 0.8887   Mean   : 77.66  
##  3rd Qu.:16.08   3rd Qu.:16.25   3rd Qu.: 0.1260   3rd Qu.:108.03  
##  Max.   :55.00   Max.   :80.00   Max.   :35.0000   Max.   :368.62  
##  NA's   :646     NA's   :284     NA's   :1056      NA's   :293     
##       DSDI               CMA              CAA              CDA        
##  Min.   :  0.6654   Min.   : 16.11   Min.   : 3.151   Min.   : 17.79  
##  1st Qu.:  0.9329   1st Qu.: 58.12   1st Qu.:23.483   1st Qu.: 80.08  
##  Median :  1.0000   Median : 64.83   Median :27.066   Median : 86.92  
##  Mean   :  3.7840   Mean   : 64.58   Mean   :27.332   Mean   : 88.09  
##  3rd Qu.:  1.1157   3rd Qu.: 71.52   3rd Qu.:31.122   3rd Qu.: 94.37  
##  Max.   :269.8500   Max.   :148.96   Max.   :74.262   Max.   :160.74  
##  NA's   :737        NA's   :443      NA's   :438      NA's   :439     
##       MDL              DDL              ...61            CH         
##  Min.   :0.0864   Min.   :0.07143   Min.   : NA    Min.   :  0.570  
##  1st Qu.:0.2632   1st Qu.:0.23739   1st Qu.: NA    1st Qu.:  7.707  
##  Median :0.4167   Median :0.33333   Median : NA    Median : 18.405  
##  Mean   :0.4079   Mean   :0.36248   Mean   :NaN    Mean   : 28.030  
##  3rd Qu.:0.5405   3rd Qu.:0.48603   3rd Qu.: NA    3rd Qu.: 40.862  
##  Max.   :1.0638   Max.   :1.11111   Max.   : NA    Max.   :145.550  
##  NA's   :590      NA's   :191       NA's   :1371   NA's   :7        
##    TransvUndu     Interdentsulci        CTU1         Log_CBL       
##  Min.   :0.0000   Min.   :0.0000   Min.   : 0.0   Min.   :-0.9676  
##  1st Qu.:0.0000   1st Qu.:0.0000   1st Qu.: 0.0   1st Qu.: 1.4545  
##  Median :1.0000   Median :0.0000   Median : 2.0   Median : 2.2976  
##  Mean   :0.5149   Mean   :0.4187   Mean   : 1.5   Mean   : 2.1924  
##  3rd Qu.:1.0000   3rd Qu.:1.0000   3rd Qu.: 3.0   3rd Qu.: 2.9848  
##  Max.   :1.0000   Max.   :1.0000   Max.   :10.0   Max.   : 3.9982  
##  NA's   :969      NA's   :996      NA's   :969    NA's   :3        
##     Log_CBW            Log_AL           Log_CBR           Log_CHR       
##  Min.   :-0.6162   Min.   :-0.5978   Min.   :-1.3863   Min.   :-0.9163  
##  1st Qu.: 0.8329   1st Qu.: 2.5177   1st Qu.:-0.7769   1st Qu.: 0.4912  
##  Median : 1.8083   Median : 3.3952   Median :-0.6125   Median : 0.6461  
##  Mean   : 1.7243   Mean   : 3.1984   Mean   :-0.5745   Mean   : 0.6339  
##  3rd Qu.: 2.5975   3rd Qu.: 4.0101   3rd Qu.:-0.3718   3rd Qu.: 0.7846  
##  Max.   : 3.8836   Max.   : 5.0294   Max.   : 0.7812   Max.   : 1.4404  
##  NA's   :69        NA's   :338       NA's   :81        NA's   :14       
##     Log_MCL          Log_MCW           Log_MCR           Log_MDE     
##  Min.   :-1.139   Min.   :-0.0619   Min.   :-0.9570   Min.   : -Inf  
##  1st Qu.: 1.901   1st Qu.: 1.4996   1st Qu.:-0.6931   1st Qu.: -Inf  
##  Median : 2.496   Median : 2.0001   Median :-0.5543   Median : -Inf  
##  Mean   : 2.336   Mean   : 1.9078   Mean   :-0.5228   Mean   : -Inf  
##  3rd Qu.: 2.889   3rd Qu.: 2.3943   3rd Qu.:-0.3830   3rd Qu.:2.176  
##  Max.   : 3.614   Max.   : 3.4078   Max.   : 0.2462   Max.   :4.067  
##  NA's   :876      NA's   :906       NA's   :907       NA's   :1044   
##     Log_MSL          Log_MEC         Log_LAF         Log_LIF     
##  Min.   :0.3507   Min.   : -Inf   Min.   : -Inf   Min.   : -Inf  
##  1st Qu.:2.8650   1st Qu.:4.230   1st Qu.: -Inf   1st Qu.: -Inf  
##  Median :3.3331   Median :4.605   Median : -Inf   Median : -Inf  
##  Mean   :3.2569   Mean   : -Inf   Mean   : -Inf   Mean   : -Inf  
##  3rd Qu.:3.7843   3rd Qu.:4.605   3rd Qu.: -Inf   3rd Qu.: -Inf  
##  Max.   :4.8173   Max.   :4.734   Max.   :2.708   Max.   :2.708  
##  NA's   :1025     NA's   :1031    NA's   :739     NA's   :755    
##     Log_DMT           Log_DDT           Log_DLAT          Log_DLIT      
##  Min.   :-2.3026   Min.   :-2.3026   Min.   :-2.3026   Min.   :-2.3026  
##  1st Qu.: 0.3365   1st Qu.: 0.2223   1st Qu.: 0.0000   1st Qu.: 0.0721  
##  Median : 0.7885   Median : 1.0986   Median : 0.8755   Median : 0.7885  
##  Mean   : 0.7166   Mean   : 0.7985   Mean   : 0.5680   Mean   : 0.5162  
##  3rd Qu.: 1.5007   3rd Qu.: 1.4478   3rd Qu.: 1.3863   3rd Qu.: 1.2074  
##  Max.   : 2.1401   Max.   : 2.3341   Max.   : 2.0968   Max.   : 2.0732  
##  NA's   :1324      NA's   :1324      NA's   :1322      NA's   :1324     
##      Log_CA         Log_CA2           Log_MA          Log_MC     
##  Min.   :2.140   Min.   :  -Inf   Min.   :1.539   Min.   :1.548  
##  1st Qu.:4.223   1st Qu.:-3.219   1st Qu.:2.197   1st Qu.:2.225  
##  Median :4.421   Median :-2.408   Median :2.420   Median :2.485  
##  Mean   :4.272   Mean   :  -Inf   Mean   :2.498   Mean   :2.629  
##  3rd Qu.:4.457   3rd Qu.:-1.897   3rd Qu.:2.639   3rd Qu.:2.944  
##  Max.   :4.479   Max.   :-1.022   Max.   :4.094   Max.   :4.059  
##  NA's   :1004    NA's   :1182     NA's   :888     NA's   :590    
##      Log_MB          Log_DA          Log_DC          Log_DB     
##  Min.   :1.792   Min.   :1.386   Min.   : -Inf   Min.   :1.792  
##  1st Qu.:2.398   1st Qu.:2.251   1st Qu.:2.326   1st Qu.:2.442  
##  Median :2.565   Median :2.485   Median :2.708   Median :2.695  
##  Mean   :2.608   Mean   :2.527   Mean   : -Inf   Mean   :2.721  
##  3rd Qu.:2.773   3rd Qu.:2.708   3rd Qu.:3.047   3rd Qu.:2.918  
##  Max.   :3.807   Max.   :4.263   Max.   :4.248   Max.   :4.382  
##  NA's   :1015    NA's   :790     NA's   :191     NA's   :845    
##     Log_MAVG        Log_DAVG        Log_DAVG2         Log_TDD      
##  Min.   : -Inf   Min.   :0.4447   Min.   :  -Inf   Min.   :-1.609  
##  1st Qu.:2.219   1st Qu.:2.0347   1st Qu.:-2.996   1st Qu.: 3.584  
##  Median :2.485   Median :2.4423   Median :-2.278   Median : 4.040  
##  Mean   : -Inf   Mean   :2.3747   Mean   :  -Inf   Mean   : 4.039  
##  3rd Qu.:2.778   3rd Qu.:2.7879   3rd Qu.:-1.561   3rd Qu.: 4.682  
##  Max.   :4.007   Max.   :4.3820   Max.   : 3.555   Max.   : 5.910  
##  NA's   :646     NA's   :284      NA's   :1195     NA's   :293     
##     Log_DSDI          Log_CMA         Log_CAA         Log_CDA     
##  Min.   :-0.4074   Min.   :2.779   Min.   :1.148   Min.   :2.879  
##  1st Qu.:-0.0694   1st Qu.:4.062   1st Qu.:3.156   1st Qu.:4.383  
##  Median : 0.0000   Median :4.172   Median :3.298   Median :4.465  
##  Mean   : 0.1028   Mean   :4.149   Mean   :3.275   Mean   :4.467  
##  3rd Qu.: 0.1095   3rd Qu.:4.270   3rd Qu.:3.438   3rd Qu.:4.547  
##  Max.   : 5.5979   Max.   :5.004   Max.   :4.308   Max.   :5.080  
##  NA's   :737       NA's   :443     NA's   :438     NA's   :439    
##     Log_MDL           Log_DDL          Log_...61        Log_CH       
##  Min.   :-2.4493   Min.   :-2.6391   Min.   : NA    Min.   :-0.5621  
##  1st Qu.:-1.3350   1st Qu.:-1.4380   1st Qu.: NA    1st Qu.: 2.0422  
##  Median :-0.8755   Median :-1.0986   Median : NA    Median : 2.9126  
##  Mean   :-1.0193   Mean   :-1.1308   Mean   :NaN    Mean   : 2.8310  
##  3rd Qu.:-0.6152   3rd Qu.:-0.7215   3rd Qu.: NA    3rd Qu.: 3.7102  
##  Max.   : 0.0619   Max.   : 0.1054   Max.   : NA    Max.   : 4.9805  
##  NA's   :590       NA's   :191       NA's   :1371   NA's   :7        
##  Log_TransvUndu Log_Interdentsulci    Log_CTU1     
##  Min.   :-Inf   Min.   :-Inf       Min.   :  -Inf  
##  1st Qu.:-Inf   1st Qu.:-Inf       1st Qu.:  -Inf  
##  Median :   0   Median :-Inf       Median :0.6931  
##  Mean   :-Inf   Mean   :-Inf       Mean   :  -Inf  
##  3rd Qu.:   0   3rd Qu.:   0       3rd Qu.:1.0986  
##  Max.   :   0   Max.   :   0       Max.   :2.3026  
##  NA's   :969    NA's   :996        NA's   :969
```

## 2.2 Check first rows

```
head(data)
```

## 2.3 Tables

### 2.3.1 Genus / Taxa Table

```
taxa<-table(data$CladeToothtype)
data_taxa<-data.frame(taxa)
data_taxa<-data_taxa[order(data_taxa$Freq, decreasing = T), ]
data_taxa$ID<-1:nrow(data_taxa)
data_taxa
```

# 3 Data Processing

## 3.1 Removing columns that have more more missing value

```
# Count the number of missing values in each column
missing_counts <- colSums(is.na(data))

# Remove columns with more than 15% missing values
data1_cleaned <- data[, missing_counts <= nrow(data)*0.15]
```

## 3.2 Removing rows with NA values

```
# Remove rows with any NA values
data1_cleaned <- na.omit(data1_cleaned)
data1_cleaned
```

## 3.3 Subtracting the observations for the Taxa that have least observations

```
lennn<-(ncol(data1_cleaned)-2)/2
taxa1<-table(data1_cleaned$CladeToothtype)
data1_cleanedd<-data.frame(taxa1)
data1_cleanedd<-data1_cleanedd[order(data1_cleanedd$Freq, decreasing = T), ]
data1_cleanedd$ID<-1:nrow(data1_cleanedd)
data1_cleanedd$CladeToothtype<-data1_cleanedd$Var1

data1_cleaned1<-data1_cleanedd[data1_cleanedd$Freq>lennn,]

data1_cleaned<-data1_cleaned[data1_cleaned$CladeToothtype%in%unique(data1_cleaned1$CladeToothtype),]

summary(data1_cleaned)
```

```
##                                  CladeToothtype    Epoch          
##  Dromaeosauridae Lateral                :295    Length:1119       
##  Tyrannosauridae Lateral                :175    Class :character  
##  Troodontidae Lateral                   : 80    Mode  :character  
##  Carcharodontosauridae Lateral          : 74                      
##  Abelisauridae Lateral                  : 63                      
##  Non-spinosaurid Megalosauroidea Lateral: 63                      
##  (Other)                                :369                      
##       CBL             CBW              CBR              CHR       
##  Min.   : 1.38   Min.   : 0.600   Min.   :0.2500   Min.   :0.400  
##  1st Qu.: 4.89   1st Qu.: 2.300   1st Qu.:0.4529   1st Qu.:1.639  
##  Median :10.91   Median : 5.810   Median :0.5287   Median :1.910  
##  Mean   :14.87   Mean   : 9.181   Mean   :0.5803   Mean   :1.924  
##  3rd Qu.:21.27   3rd Qu.:13.745   3rd Qu.:0.6659   3rd Qu.:2.185  
##  Max.   :54.50   Max.   :48.600   Max.   :2.1840   Max.   :3.575  
##                                                                   
##        DC             DDL                CH             Log_CBL      
##  Min.   : 4.50   Min.   :0.08333   Min.   :  2.200   Min.   :0.3221  
##  1st Qu.:10.00   1st Qu.:0.25000   1st Qu.:  8.835   1st Qu.:1.5872  
##  Median :15.00   Median :0.33333   Median : 19.740   Median :2.3897  
##  Mean   :17.25   Mean   :0.36692   Mean   : 29.830   Mean   :2.3152  
##  3rd Qu.:20.00   3rd Qu.:0.50000   3rd Qu.: 43.420   3rd Qu.:3.0571  
##  Max.   :60.00   Max.   :1.11111   Max.   :145.550   Max.   :3.9982  
##                                                                      
##     Log_CBW           Log_CBR           Log_CHR            Log_DC     
##  Min.   :-0.5108   Min.   :-1.3863   Min.   :-0.9163   Min.   :1.504  
##  1st Qu.: 0.8329   1st Qu.:-0.7920   1st Qu.: 0.4942   1st Qu.:2.303  
##  Median : 1.7596   Median :-0.6374   Median : 0.6471   Median :2.708  
##  Mean   : 1.7192   Mean   :-0.5951   Mean   : 0.6312   Mean   :2.721  
##  3rd Qu.: 2.6207   3rd Qu.:-0.4067   3rd Qu.: 0.7818   3rd Qu.:2.996  
##  Max.   : 3.8836   Max.   : 0.7812   Max.   : 1.2740   Max.   :4.094  
##                                                                       
##     Log_DDL            Log_CH      
##  Min.   :-2.4849   Min.   :0.7885  
##  1st Qu.:-1.3863   1st Qu.:2.1787  
##  Median :-1.0986   Median :2.9826  
##  Mean   :-1.1112   Mean   :2.9528  
##  3rd Qu.:-0.6931   3rd Qu.:3.7709  
##  Max.   : 0.1054   Max.   :4.9805  
##
```

## 3.4 Spliting the Log variables and the original

```
# Select variables that contain "log" and the first column
selected_cols <- c(1,2, grep("Log", names(data1_cleaned)))

# Subset the data frame
data_log <- data1_cleaned[, selected_cols]

data_log
```

```
names(data_log)[-1]  <- gsub(" ", "_", names(data_log)[-1] )


# Identify columns that contain "log"
log_cols <- grep("Log", names(data1_cleaned))

# Include the first column
cols_to_keep <- setdiff(1:ncol(data1_cleaned), log_cols)

# Ensure the first column is included
cols_to_keep <- union(1, cols_to_keep)

# Subset the data frame
data_original <- data1_cleaned[, cols_to_keep]

data_original
```

# 4 Visualization

## 4.1 Original data

```
correlation_matrix <- cor(data_original[, -c(1, 2)])
# Plot correlation matrix
corrplot(correlation_matrix, method = "color", type = "lower", 
         addCoef.col = "black", 
         tl.col = "black", 
         tl.srt = 45, 
         diag = FALSE, 
         order = "hclust", 
         col = colorRampPalette(c("blue", "white", "red"))(200))
```

## 4.2 Log data

```
correlation_matrix <- cor(data_log[, -c(1, 2)])
# Plot correlation matrix
corrplot(correlation_matrix, method = "color", type = "lower", 
         addCoef.col = "black", 
         tl.col = "black", 
         tl.srt = 45, 
         diag = FALSE, 
         order = "hclust", 
         col = colorRampPalette(c("blue", "white", "red"))(200))
```

```
colnames(data_original)
```

```
## [1] "CladeToothtype" "Epoch"          "CBL"            "CBW"           
## [5] "CBR"            "CHR"            "DC"             "DDL"           
## [9] "CH"
```

```
# Filter out taxa with less than 10 observations
data_log <- data_log %>%
  group_by(CladeToothtype) %>%                   # Group by 'Taxa' column
  filter(n() >= 10) %>%                # Keep only groups with 10 or more observations
  ungroup()                            # Ungroup after filtering
# Filter out taxa with less than 10 observations
data_original <- data_original %>%
  group_by(CladeToothtype) %>%                   # Group by 'Taxa' column
  filter(n() >= 10) %>%                # Keep only groups with 10 or more observations
  ungroup() 
# Get the count of each unique value in the column
category_counts <- table(data_log$CladeToothtype)

# Filter unique values that have more than 0 observations
unique_values <- names(category_counts[category_counts > 0])

# Print the result
#print(unique_values)
```

# 5 Merging data clade with tooth to add more classes

```
data1$CladeToothtype<-ifelse(!(data1$CladeToothtype%in%unique_values),data1$Clade,data1$CladeToothtype)

data1<-data1[,-c(1,2,3,4,6:14,16:19)]#until 19
#clade: 4, taxa:2, teethtaxa: 3, cladetteth: 5, epoch:15
#data<-inner_join(dd,data)
#data<-data[!duplicated(data),]

data1$`TransvUndu`<-ifelse(data1$`Transv. Undu.`!=0 & !is.na(data1$`Transv. Undu.`),1,data1$`Transv. Undu.`)

data1$`Interdentsulci`<-ifelse(data1$`Interdent. sulci`!=0 & !is.na(data1$`Interdent. sulci`),1,data1$`Interdent. sulci`)

data1$LAF<-ifelse(data1$LAF=="6-7",6.5,data1$LAF)

data1$CTU1 <- sub(".*?(\\d+).*", "\\1", data1$CTU)

data1<- data1 %>% select(-CTU,-`Interdent. sulci`,-`Transv. Undu.`)

# Convert columns to numeric, then create log-transformed columns
data1 <- data1 %>%
  mutate(across(3:ncol(data1), as.numeric)) %>%
  mutate(across(3:ncol(data1), log, .names = "Log_{.col}"))
```

```
## Warning: There were 17 warnings in `mutate()`.
## The first warning was:
## ℹ In argument: `across(3:ncol(data1), as.numeric)`.
## Caused by warning:
## ! NAs introduced by coercion
## ℹ Run `dplyr::last_dplyr_warnings()` to see the 16 remaining warnings.
```

```
## Warning: There were 3 warnings in `mutate()`.
## The first warning was:
## ℹ In argument: `across(3:ncol(data1), log, .names = "Log_{.col}")`.
## Caused by warning:
## ! NaNs produced
## ℹ Run `dplyr::last_dplyr_warnings()` to see the 2 remaining warnings.
```

```
data1$CladeToothtype<-as.factor(data1$CladeToothtype)
#data1$Epoch<-ifelse(data1$Epoch=="'Middle Cretaceous'","Middle Cretaceous", data1$Epoch)
#data1$Epoch<-as.factor(data1$Epoch)


#data$Taxa<-as.factor(paste0(data$`Taxa (Genus)`,data$Maturity,sep=" "))
data1<-data.frame(data1)


# Count the number of missing values in each column
missing_counts <- colSums(is.na(data1))

# Remove columns with more than 15% missing values
data1_cleaned <- data1[, missing_counts <= nrow(data1)*0.15]

# Remove rows with any NA values
data1_cleaned <- na.omit(data1_cleaned)
data1_cleaned
```

```
lennn<-(ncol(data1_cleaned)-2)/2
taxa1<-table(data1_cleaned$CladeToothtype)
data1_cleanedd<-data.frame(taxa1)
data1_cleanedd<-data1_cleanedd[order(data1_cleanedd$Freq, decreasing = T), ]
data1_cleanedd$ID<-1:nrow(data1_cleanedd)
data1_cleanedd$CladeToothtype<-data1_cleanedd$Var1

data1_cleaned1<-data1_cleanedd[data1_cleanedd$Freq>lennn,]

data1_cleaned<-data1_cleaned[data1_cleaned$CladeToothtype%in%unique(data1_cleaned1$CladeToothtype),]

summary(data1_cleaned)
```

```
##                                  CladeToothtype    Epoch          
##  Dromaeosauridae Lateral                :295    Length:1119       
##  Tyrannosauridae Lateral                :175    Class :character  
##  Troodontidae Lateral                   : 80    Mode  :character  
##  Carcharodontosauridae Lateral          : 74                      
##  Abelisauridae Lateral                  : 63                      
##  Non-spinosaurid Megalosauroidea Lateral: 63                      
##  (Other)                                :369                      
##       CBL             CBW              CBR              CHR       
##  Min.   : 1.38   Min.   : 0.600   Min.   :0.2500   Min.   :0.400  
##  1st Qu.: 4.89   1st Qu.: 2.300   1st Qu.:0.4529   1st Qu.:1.639  
##  Median :10.91   Median : 5.810   Median :0.5287   Median :1.910  
##  Mean   :14.87   Mean   : 9.181   Mean   :0.5803   Mean   :1.924  
##  3rd Qu.:21.27   3rd Qu.:13.745   3rd Qu.:0.6659   3rd Qu.:2.185  
##  Max.   :54.50   Max.   :48.600   Max.   :2.1840   Max.   :3.575  
##                                                                   
##        DC             DDL                CH             Log_CBL      
##  Min.   : 4.50   Min.   :0.08333   Min.   :  2.200   Min.   :0.3221  
##  1st Qu.:10.00   1st Qu.:0.25000   1st Qu.:  8.835   1st Qu.:1.5872  
##  Median :15.00   Median :0.33333   Median : 19.740   Median :2.3897  
##  Mean   :17.25   Mean   :0.36692   Mean   : 29.830   Mean   :2.3152  
##  3rd Qu.:20.00   3rd Qu.:0.50000   3rd Qu.: 43.420   3rd Qu.:3.0571  
##  Max.   :60.00   Max.   :1.11111   Max.   :145.550   Max.   :3.9982  
##                                                                      
##     Log_CBW           Log_CBR           Log_CHR            Log_DC     
##  Min.   :-0.5108   Min.   :-1.3863   Min.   :-0.9163   Min.   :1.504  
##  1st Qu.: 0.8329   1st Qu.:-0.7920   1st Qu.: 0.4942   1st Qu.:2.303  
##  Median : 1.7596   Median :-0.6374   Median : 0.6471   Median :2.708  
##  Mean   : 1.7192   Mean   :-0.5951   Mean   : 0.6312   Mean   :2.721  
##  3rd Qu.: 2.6207   3rd Qu.:-0.4067   3rd Qu.: 0.7818   3rd Qu.:2.996  
##  Max.   : 3.8836   Max.   : 0.7812   Max.   : 1.2740   Max.   :4.094  
##                                                                       
##     Log_DDL            Log_CH      
##  Min.   :-2.4849   Min.   :0.7885  
##  1st Qu.:-1.3863   1st Qu.:2.1787  
##  Median :-1.0986   Median :2.9826  
##  Mean   :-1.1112   Mean   :2.9528  
##  3rd Qu.:-0.6931   3rd Qu.:3.7709  
##  Max.   : 0.1054   Max.   :4.9805  
##
```

```
# Select variables that contain "log" and the first column
selected_cols <- c(1,2, grep("Log", names(data1_cleaned)))

# Subset the data frame
data_log <- data1_cleaned[, selected_cols]

data_log
```

```
names(data_log)[-1]  <- gsub(" ", "_", names(data_log)[-1] )


# Identify columns that contain "log"
log_cols <- grep("Log", names(data1_cleaned))

# Include the first column
cols_to_keep <- setdiff(1:ncol(data1_cleaned), log_cols)

# Ensure the first column is included
cols_to_keep <- union(1, cols_to_keep)

# Subset the data frame
data_original <- data1_cleaned[, cols_to_keep]

data_original
```

```
colnames(data_original)
```

```
## [1] "CladeToothtype" "Epoch"          "CBL"            "CBW"           
## [5] "CBR"            "CHR"            "DC"             "DDL"           
## [9] "CH"
```

```
# Filter out taxa with less than 10 observations
data_log <- data_log %>%
  group_by(CladeToothtype) %>%                   # Group by 'Taxa' column
  filter(n() >= 10) %>%                # Keep only groups with 10 or more observations
  ungroup()                            # Ungroup after filtering
# Filter out taxa with less than 10 observations
data_original <- data_original %>%
  group_by(CladeToothtype) %>%                   # Group by 'Taxa' column
  filter(n() >= 10) %>%                # Keep only groups with 10 or more observations
  ungroup() 
# Get the count of each unique value in the column
category_counts <- table(data_log$CladeToothtype)

# Filter unique values that have more than 0 observations
unique_values1 <- names(category_counts[category_counts > 0])
```

# 6 Saving the clean dataset

```
write.csv(data_log,"teeth_data_log_clade_epoch1.csv", row.names = FALSE)
write.csv(data_original,"teeth_data_clade_epoch1.csv", row.names = FALSE)
```

# 7 Summary tables

```
library(readxl)
data <- read_xlsx("Crown measurement dataset Kem Kem theropods.xlsx")
```

```
## New names:
## • `` -> `...1`
## • `Position` -> `Position...7`
## • `Position` -> `Position...18`
## • `CH` -> `CH...22`
## • `CH` -> `CH...60`
## • `` -> `...61`
```

```
table(data$Country)
```

```
## 
##  Argentina  Australia     Brazil     Canada      China     France    Germany 
##         99         17          1        300         89         50         14 
##      India      Italy      Japan Madagascar   Mongolia    Morocco      Niger 
##         11          3         15         78         50         70         24 
##   Portugal        RSA       U.K.       U.S.    Uruguay        USA 
##         23          2         57        435          2         31
```

```
table(data$Maturity)
```

```
## 
##                               /                               ? 
##                               1                             570 
##                           Adult                   Adult (24 yo) 
##                             244                              10 
##             Adult or near adult               Adult or subadult 
##                              53                              13 
##                  Adult; Stage 4                          Adult? 
##                              80                             122 
##               At least subadult                   Fairly mature 
##                               2                              26 
##                       Hatchling                        Immature 
##                               3                              22 
##             Immature (juvenile)                Immature (young) 
##                              29                              16 
##       Immature (young); Stage 1   Immature approaching maturity 
##                              11                               2 
##               Immature; Stage 2                       Immature? 
##                               6                              34 
##                        Juvenile            Juvenile or subadult 
##                               6                               1 
##               Juvenile/immature  Juvenile; Small Stage 1 (7 yo) 
##                              25                               9 
##       Late juvenile or subadult       No indication of maturity 
##                               9                               7 
##                       Old adult               Old adult (12 yo) 
##                               1                               6 
##               Old adult (28 yo)                        Subadult 
##                              22                              25 
## Subadult; Large Stage 1 (18 yo)                       Subadult? 
##                               6                               3 
##                           Young                  Young subadult 
##                               3                               4
```

```
table(data$Epoch)
```

```
## 
##              'Middle Cretaceous'                 Early Cretaceous 
##                              238                               81 
##                   Early Jurassic                  Late Cretaceous 
##                                9                              724 
##                    Late Jurassic Late Jurassic - Early Cretaceous 
##                              205                                2 
##                    Late Triassic                  Middle Jurassic 
##                               57                               55
```
