# Supplementary material for: Enhancing the classification of isolated theropod teeth using machine learning: a comparative study
Source: PeerJ. 2025 Mar 26;13:e19116. doi: 10.7717/peerj.19116 (PMC11954464; doi:10.7717/peerj.19116)
Supplement: Supplemental Information 2 [file peerj-13-19116-s002.zip › data_processing_new_isolated_theropod_teeth.html]

New isolated theropod teeth


Code 

- Show All Code
- Hide All Code

# New isolated theropod teeth

#### Carolina Marques

#### 2025-01-07

```
library(readxl)
library(dplyr)
```

```
## 
## Attaching package: 'dplyr'
```

```
## The following objects are masked from 'package:stats':
## 
##     filter, lag
```

```
## The following objects are masked from 'package:base':
## 
##     intersect, setdiff, setequal, union
```

```
data <- read_xlsx("New_theropod_teeth.xlsx")#reading data
data<-data[,c(1:15)]
# Convert columns to numeric, then create log-transformed columns
data <- data %>%
  mutate(across(9:ncol(data), as.numeric)) %>%
  mutate(across(9:ncol(data), log, .names = "Log_{.col}"))

write.csv(data,"new_theropod_teeth_data.csv", row.names = FALSE)
```
